# Triparental inheritance in *Dictyostelium*

**DOI:** 10.1073/pnas.1814425116

**Published:** 2019-01-22

**Authors:** Gareth Bloomfield, Peggy Paschke, Marina Okamoto, Tim J. Stevens, Hideko Urushihara

**Affiliations:** ^a^Division of Cell Biology, Medical Research Council Laboratory of Molecular Biology, Cambridge Biomedical Campus, Cambridge, CB2 0QH, United Kingdom;; ^b^Faculty of Life and Environmental Sciences, University of Tsukuba, Tsukuba, 305-8572 Ibaraki, Japan

**Keywords:** social amoebae, syngamy, meiosis, recombination, mitochondria

## Abstract

Sex promotes the recombination and reassortment of genetic material and is prevalent across eukaryotes, although our knowledge of the molecular details of sexual inheritance is scant in several major lineages. In social amoebae, sex involves a promiscuous mixing of cytoplasm before zygotes consume the majority of cells, but for technical reasons, sexual progeny have been difficult to obtain and study. We report here genome-wide characterization of meiotic progeny in *Dictyostelium discoideum*. We find that recombination occurs at high frequency in pairwise crosses between all three mating types, despite the absence of the Spo11 enzyme that is normally required to initiate crossover formation. Fusions of more than two gametes to form transient syncytia lead to frequent triparental inheritance, with haploid meiotic progeny bearing recombined nuclear haplotypes from two parents and the mitochondrial genome from a third. Cells that do not contribute genetically to the *Dictyostelium* zygote nucleus thereby have a stake in the next haploid generation. *D. discoideum* mitochondrial genomes are polymorphic, and our findings raise the possibility that some of this variation might be a result of sexual selection on genes that can promote the spread of individual organelle genomes during sex. This kind of self-interested mitochondrial behavior may have had important consequences during eukaryogenesis and the initial evolution of sex.

## Significance

Sex produces a new individual in which genetic material is reassorted and recombined. Most often, nuclear DNA is inherited from two parents, and organelle genomes are transmitted from only one of those parents. We report here genome-wide analysis of sexual recombination in social amoebae and show that inheritance in this protozoan is often triparental. Unusually, more than two gametes frequently fuse together and then split apart, allowing cytoplasm from multiple parents to be mixed. Progeny produced after two nuclei fuse and undergo meiosis thereby inherit nuclear DNA from two parents, and often mitochondrial DNA from a third. Our findings raise questions about mechanisms by which mitochondrial genes can promote self-interested organelle behavior during sex in social amoebae and other eukaryotes.

Inheritance of nuclear genes largely follows the Mendelian laws of segregation and independent assortment. Individual eukaryotes are produced either clonally, having a single parent, or sexually, with two parents. In contrast, cytoplasmic genes display non-Mendelian patterns of inheritance and are typically transmitted only from one of the two parents during sex. The uncoupled inheritance of these different sets of genes creates recurrent genetic conflict (1–3). The effects of such conflict are now well understood in diverse animals and plants, but in many less well-studied eukaryotes, the effects of sexual conflict are unclear. Social amoebae present intriguing examples of conflict (4). These protists proliferate as single cells, feeding on bacteria, but when starved, they undergo developmental programs involving intimate contacts between conspecific cells. In their asexual cycle, many thousands of social amoebae aggregate to form multicellular structures, ultimately forming fruiting bodies consisting of many individual haploid spore cells atop slender stalks. In most genera, the stalk is composed of dead, vacuolated cells; this leads to the potential for conflict, as any variant that is overrepresented among spore cells is at an advantage (4). The sexual cycle of social amoebae is even more conflictual: as they differentiate, zygotes feed cannibalistically on surrounding cells, including other zygotes as well as haploid amoebae (4, 5).

**Fig. 1.**
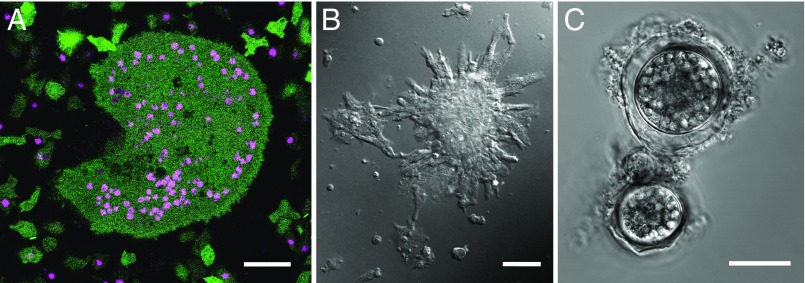
Sexual development in *Dictyostelium*. (*A*) A syncytium formed after fusion of a *D. discoideum* strain (HM1558) expressing cytoplasmic GFP with a strain (AX2) expressing RFP-tagged histone 2B, surrounded by smaller fused and unfused amoebae. The cells were made fusion competent separately and then mixed and shaken together for 1 h before incubation in a chamber slide. These cells were imaged 7 h after mixing. (*B*) Aggregation around zygotes: cells of strain AC4 were made fusion competent and incubated in a chamber slide. Several zygotes as well as a haploid amoebae aggregate around the large zygote in the center-right of the field. (*C*) Mature macrocysts 15 d after mixing strains HM597 and HM598; cannibalized amoebae are still prominent in food vacuoles inside the walled cysts. (Scale bar, 25 µm.)

Sex in social amoebae has a number of other unusual features. Several species have more than two mating types (6, 7), which are specified in part by homeodomain-like proteins in *Dictyostelium* (8). Social amoeba gametes are indistinguishable in size (9), and their fusion is reliant on HAP2/GCS1-related proteins (10). Gamete fusion in *Dictyostelium* is unusually promiscuous, with no mechanism preventing multiple gametes from fusing, so that syncytia can form (Fig. 1*A*) (11–13). This is unusual: across other eukaryotes, syngamy is typically under tight control to ensure strict genome doubling because accidental polyspermy leads to polyploidy (14, 15). *Dictyostelium* sexual syncytia break apart gradually over the course of several hours, giving rise to binucleate cells before nuclear fusion occurs (13); control of nuclear pairing and fusion is not understood in these cells. Uninucleate zygotes then attract and ingest surrounding cells (Fig. 1*B*) and grow without undergoing mitosis to form semidormant walled diploid cells called macrocysts (Fig. 1*C*). Meiosis is believed to occur in young macrocysts, where there is ultrastructural evidence for synaptonemal complexes (12, 16). Macrocysts can remain dormant for several weeks before germinating to release haploid amoebae, but the triggers for germination remain unclear, so generating progeny in the laboratory is difficult. In one cross in which recombinant *D. discoideum* haploid progeny could be obtained, crossovers on one chromosome were found to be as frequent as in *Saccharomyces cerevisiae*, which has one of the highest crossover frequencies known (17, 18). In retrospect, this is surprising: genome sequencing later revealed that although a number of meiosis-specific genes are conserved in social amoebae, the gene encoding Spo11, the transesterase normally required to initiate formation of chiasmata (19) has been lost in this lineage (20). How recombination might occur in the absence of this key enzyme remains mysterious (21, 22).

## Results and Discussion

**Fig. 2.**
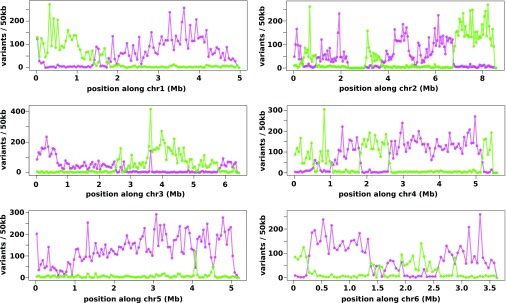
Recombination in one representative progeny clone. After whole-genome sequencing of XGB1, a haploid progeny clone from the cross between HM597 and HM598, variants specific to each parent were tallied in 50-kb segments along all six nuclear chromosomes. Variants from HM597 are plotted in magenta; those from HM598 are plotted in green. *D. discoideum* chromosomes are telocentric, and centromeres are plotted to the *Left*. Errors in read mapping and variant-calling lead to some noise in these data, giving rise to the low-level minority counts where the other parent’s variants predominate. A region of chromosome 2 in the plots has no apparent variants because a duplication in the assembly has been masked.

**Fig. 3.**
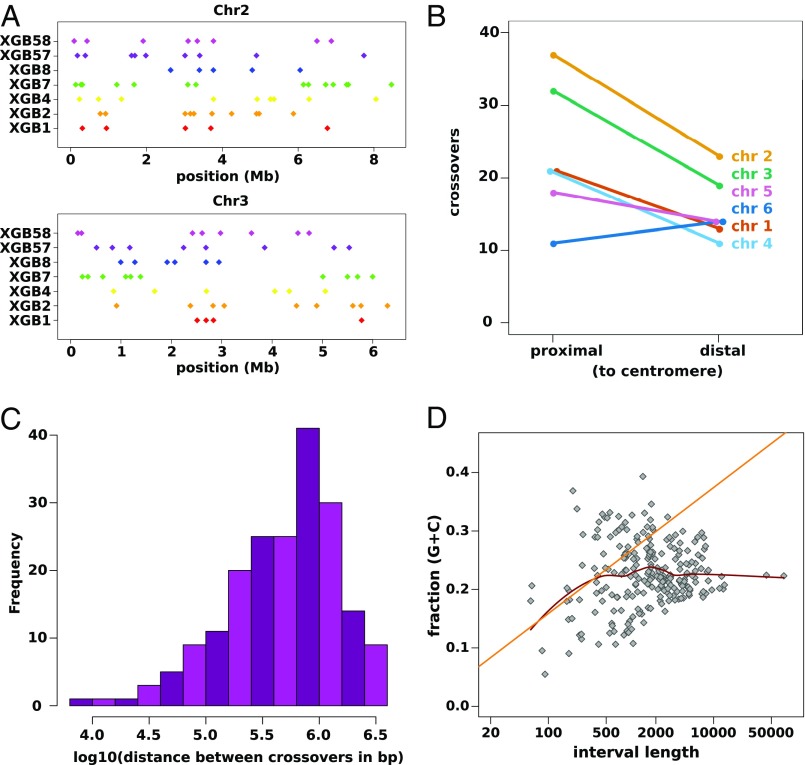
Overall patterns of recombination. (*A*) The positions of crossovers along the two longest chromosomes (2 and 3) are displayed for all seven sequenced progeny clones. (*B*) In five of six chromosomes, there are more crossovers overall in the centromere-proximal than the centromere-distal half. (*C*) Crossovers tend to be spaced hundreds of kilobases apart, although a small fraction clusters more closely. More progeny will be required to assess confidently the nature of crossover interference in social amoebae. (*D*) Plotting the G+C base composition of the nucleotide sequences between the variants flanking each crossover suggests a bias in crossover designation toward low G+C regions. Longer intervals by definition give less precise bounds on the true crossover site, and with increasing length merely tend to approximate the overall genomic G+C composition; the true average composition should be approximated by the y-intercept. The straight line represents a linear model fit on the intervals shorter than 300 bp, whereas the curve represents a quadratic polynomial Loess least-squares fit using all intervals.

To investigate these inheritance patterns in greater detail, we germinated macrocysts from three pairwise crosses between strains of all three *D. discoideum* mating types (HM597, HM598, and WS2162, which belong to mating types II, I, and III, respectively) and sequenced the genomes of haploid progeny clones along with those of their parents. The parental genomes were extensively polymorphic, so that after filtering of putative variants, thousands of sites could be used to assess recombination in meiotic progeny across the 34-Mb genome in each cross. The type II parent strain, HM597, is a cycloheximide-resistant mutant; initial observations confirmed that *cycA*, which lies on chromosome 1 (23), and the mating-type locus, on chromosome 5 (6), were reassorted in several putative meiotic progeny (we refer to haploid parents and haploid progeny in this system because the diploid phase is reduced, only existing for a brief period before meiosis). We identified a mutation in the *rpl36a* gene on chromosome 1 that cosegregates with, and very likely causes, cycloheximide resistance (*Material and Methods*). Genome-wide analysis of progeny from these three crosses revealed that recombination is very frequent, with at least one crossover per chromosome (Fig. 2 and *SI Appendix*, Fig. S1). We found that progeny from each macrocyst are identical, implying that only one meiotic product survives in each cyst, as has been reported previously in some *Dictyostelium* species (24, 25) (*SI Appendix*, Table S1). However, different macrocysts in a given cross give rise to diverse progeny genotypes (Fig. 3 and *SI Appendix*, Figs. S1 and S2).

We reasoned that the unusual Spo11-independent recombination during sex in social amoebae could be connected with the conflictual nature of macrocyst formation, during which large numbers of “victim” amoebae are consumed (22); more broadly, conflict is thought to have had pervasive roles during the evolution of meiosis (26). We hypothesized that transmission of genes from consumed cells into zygotes, and thence the next generation, could be an evolutionary incentive that might help explain their participation in the process, resulting in some modification to the normal process of recombination. To test this idea, we performed a three-way cross in which the mating type I and II strains used in the crosses here were mixed with an excess of *matA* null cells. These mutant cells are able to fuse with other cells and to form stable parasexual diploids, but are unable to contribute to form fully developed macrocysts when mixed with cells of any mating type, and so are unable to produce meiotic progeny (8) (*SI Appendix*, Fig. S3). In this three-way cross, any lateral genetic contribution from the *matA* mutant cells into progeny can be detected by tracking sequence variants present solely in the mutant background.

Again, similarly to the two-way crosses, recombination was very frequent in progeny from a three-way cross. (Fig. 3*A* and *SI Appendix*, Fig. S2). Across all progeny characterized in two- and three-way crosses, crossovers occurred on average approximately once per megabase in each meiosis (genetic sizes of chromosomes from 394 to 895 cM; 33.5 M in total across the 34-Mb genome), comparable in frequency with budding and fission yeasts (18). Our genome-wide analysis of meiotic progeny in *Dictyostelium* therefore confirms that high-frequency recombination occurs in social amoebae in the absence of Spo11.

*D. discoideum* chromosomes are telocentric, and crossovers occurred more often in the centromere-proximal half than the distal half in five of six chromosomes (Fig. 3*B*). The single chromosome in which this pattern was not observed, chromosome six, is the shortest in this species, suggesting that physical distance from the centromere affects the likelihood of crossover formation. Crossovers were often regularly spaced 500–1,000 kb apart, but were sometimes very closely spaced, within 50 kb of each other, suggesting that an interference-independent pathway operates in this species (Fig. 3*C*). Where flanking variants were near enough that crossover sites could be predicted with some accuracy, they could mostly be mapped within noncoding sequence, with a bias toward more A/T-rich sequences (Fig. 3*D* and *SI Appendix*, Table S2).

Ribosomal DNA, which is maintained in growing cells as multiple copies of a linear extrachromosomal element, was inherited largely uniparentally (*SI Appendix*, Fig. S4). In *Dictyostelium*, the rDNA is maintained at high copy number as extrachromosomal palindromic molecules, and a chromosomal copy has been proposed to exist on chromosome 4 (27, 28), but has not been mapped precisely because it appears to be flanked by repetitive sequences. Our data are consistent with a postmeiotic regeneration of extrachromosomal rDNA; across the progeny we have sequenced so far, rDNA genotype segregates with three chromosomal intervals, none of which lies within chromosome 4. One of these intervals, on chromosome 2, is contiguous in the current assembly, and can be ruled out as a location for a putative template. Another cosegregating chromosomal 2 interval, approximately between the genes *pyr1-3* and *DDB_G0276527*, is not currently contiguous. However, because an unmapped contig containing the full rDNA sequence was previously assembled from a collection of reads enriched in sequences from chromosomes 4 and 5 (28), the likelier candidate is a noncontiguous region of chromosome 5, approximately between genes *DDB_G0288215* and *DDB_G0288627*, that also cosegregates with rDNA genotype in progeny. Longer sequencing reads will be required to map chromosomal rDNA sequences accurately. Finally, and also relating to extrachromosomal nuclear DNA elements, one of the parental strains (WS2162) carries a high-copy nuclear plasmid that is stably carried by haploid cells during proliferation and asexual development (29); all progeny from crosses involving this strain lacked detectable plasmid, as assessed by whole-genome sequencing and PCR (*SI Appendix*, Fig. S5).

**Fig. 4.**
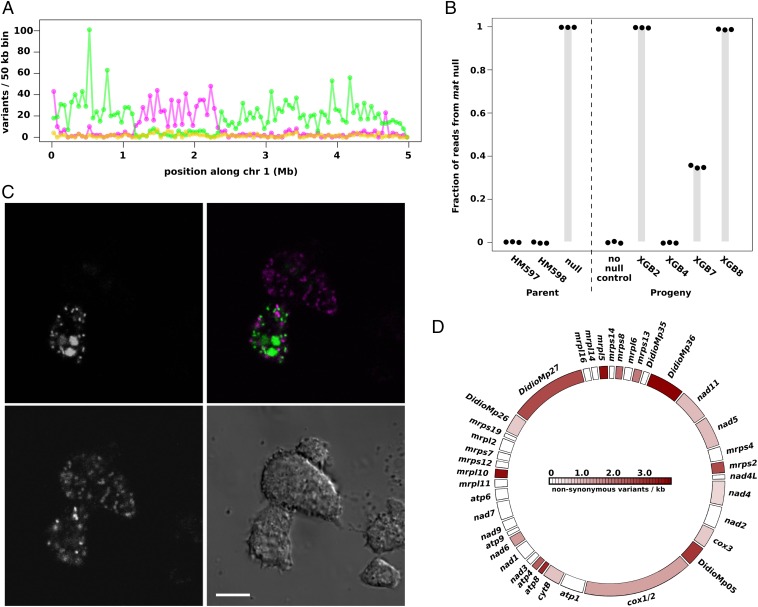
Lateral transmission of mitochondria in the *Dictyostelium* sexual cycle. (*A*) We have so far found no evidence for transmission of nuclear DNA from the mutant parent in a three-way cross between HM597, HM598, and the *matA* null strain HM1524; shown here is one representative chromosome from one progeny clone, XGB8. Variants specific to HM597 are shown in magenta, HM598 variants are in green, HM1524 (AX2) variants are in gold; all putative HM1524 variants appear to be false positives. (*B*) In contrast, lateral transfer of mitochondrial DNA clearly occurred in three of four sequenced progeny. Sequence reads specific to the *matA* null strain were counted at three positions along the mitochondrial genome for the three parent strains and one two-way progeny (“no null control”, XGB1), as well as the four three-way progeny. In the three progeny with lateral transmission of mtDNA, all other variants along the mitochondrial chromosome also showed similar contribution from the *matA* null. (*C*) Three-way fusions were directly observed between an HM598 line expressing GFP-tagged histone 2B (green, *Upper Left*), a AX2 line expressing GFP targeted to the mitochondrial matrix with the TopA N-terminal targeting motif (green, *Upper Left*), and an HM1558 line expressing RFP anchored on the mitochondrial outer membrane using the GemA C-terminal targeting motif (magenta, *Lower Left*). (*Lower Right*) A DIC image is shown. Mitochondria were mixed in the syncytial cytoplasm and were not observed to undergo fusion. (Scale bar, 10 µm.) (*D*) The *D. discoideum* mitochondrial genome is unevenly polymorphic: the density of nonsynonymous polymorphisms in protein coding genes across strains used in this study is shown (homing endonuclease genes are omitted). For comparison, the greenbeard genes *tgrB1* and *tgrC1* each have more than 40 polymorphisms per kilobase (31). *atp4* is within the top percentile of polymorphic genes in *D. discoideum* according to this dataset; this gene, along with *atp8*, was found to have the highest dN/dS ratios in a comparison of mitochondrial genes in several social amoeba species (45).

Returning to the question of whether lateral transmission of genes might occur from “victims” into the zygote, we have not been able to identify any nuclear chromosomal variants that are unique to the *matA* null parent in progeny analyzed so far from three-way crosses, indicating that the frequency of lateral inheritance of nuclear haplotypes from “victims” is low in *D. discoideum*, if it occurs at all (Fig. 4*A* and *SI Appendix*, Fig. S2). Remarkably, however, in three of four sequenced progeny from three-way crosses, we found that mitochondrial genomes were inherited from the *matA* null cells (Fig. 4*B*). These haploid progeny have three genetic parents: two contributing nuclear haplotypes and a third contributing some or all mitochondrial genome copies. In two of these progeny clones, almost all mtDNA had been inherited laterally, whereas in the third, around 40% was of the *matA* null mitotype. Inheritance of the mitochondrial genome is therefore not strictly uniparental, but often one mitotype makes up by far the largest portion; we describe these as predominant mitotypes. PCRs designed to specifically amplify the *matA* null mitotype revealed that 13 of 42 independent progeny from the three-way cross inherited mtDNA laterally (*SI Appendix*, Fig. S6 and Table S1). Using fluorescent proteins targeted to mitochondria and nuclei, we could visualize thorough mixing of cytoplasms in syncytia formed during three-way crosses, indicating that lateral transmission of mitochondria likely occurs via these transient fusions early in the sexual cycle (Fig. 4*C*). We cannot exclude the possibility that it might also occur by escape of mitochondria from cannibalized cells within food vacuoles in the zygote, but presumably much less frequently than through fusion at the syncytial stage.

Our finding that one parental mitotype dominated in each progeny clone in most cases is consistent with an earlier observation in a related species (30), although in several cases we found that two mitotypes were retained in near-equal proportions (*SI Appendix*, Table S1). In the two-way HM597 × HM598 cross, 10 of 11 progeny carried predominantly the HM597 mitotype, and the other carried both mitotypes in near-equal quantities. For the three-way cross, the HM597 mitotype was predominant in 31 of 41 progeny, HM598 in five, and the *matA* null mitotype in two, whereas in three progeny, no single mitotype was predominant. In all five progeny in which the HM598 mitotype predominated, the *matA* null mitotype was present in small quantities (*SI Appendix*, Table S1). This indicates that mitochondrial inheritance is not strictly controlled by the mating-type locus in this species; similarly, biparental inheritance often occurs in myxogastrids, one of the sister groups of social amoebae (31, 32). The overrepresentation of HM597 mitotypes in progeny obtained so far suggests that mitochondrial inheritance is not random in crosses involving this strain. Further crosses and backcrosses will be needed to distinguish between effects of nuclear genotype (including mating type) versus mitotypes, as well as the effect of the *matA* null mutation on mitochondrial inheritance, if any. We note that because each *Dictyostelium* progeny clone can inherit multiple mitotypes, it is likely that some progeny have four or more genetic parents. Mitotype inheritance frequency could simply follow passively from the overall fusion frequency of each strain, but there is also clearly scope after fusion for both nuclear and mitochondrial genes to promote the inheritance of particular mitochondria. Mitochondrial variants that are relatively successful at promoting gamete fusion or subsequent survival in zygotes and transmission into haploid progeny, or both, will be expected to spread through the population, responding to a form of sexual selection. Our contrived experimental set-up involving the *matA* mutant defective in zygogenesis gives a concrete example of how this reassortment can allow mitochondria to escape from uncompetitive nuclear backgrounds, provided that cell fusion occurs.

The asexual developmental cycle of social amoebae, in which fruiting bodies are constructed with cellular stalks, also engenders conflict: variants that avoid differentiating into stalk cells can act as social parasites and spread through the population (4). We wondered whether mitochondria could spread laterally during cell fusions that occur in asexual aggregates in *Dictyostelium*. To measure this, we mixed cells containing either a fluorescent histone or mitochondrial reporter and measured the frequency of spores containing both reporters after differentiation. These dual-labeled cells could be the result of transient fusions in which mitochondria are transferred without nuclear fusion, or more stable fusions leading to nonsexual parasexual diploid cells, which occur at a frequency of 10^−6^ to 10^−5^ in similar crosses (8). We found that lateral transmission of mitochondria occurred at a frequency of around 10^−4^ to 10^−3^ (*SI Appendix*, Fig. S7). This suggests, consistent with earlier reports of transient anastomoses and transfer of cytoplasm between aggregating cells (33), that transient cell fusions are more frequent than full parasexual fusions, and allow mitochondria (and other cytoplasmic elements) to be laterally transmitted. The frequency of lateral transfer in the asexual cycle found here is relatively low, but it may provide a way for self-interested cytoplasmic genomes in some strains to escape suffering the stalk cell fate, enabling dispersal and self-perpetuation. We predict that frequencies of this asexual lateral transfer may vary in different isolates, likely influenced by nuclear as well as cytoplasmic genes.

Our findings suggest strong sexual selection on mitochondrial genotypes might occur in social amoebae. As an initial approach toward assessing the genetic effects of this proposed selection, we cataloged the mitochondrial genome variants of the four parental strains of this study (the three wild-type parental strains plus the *matA* mutant), as well as 20 other *D. discoideum* isolates for which whole-genome data are available (34). The mitochondrial genes of these isolates are very variable in their degree of polymorphism (Fig. 4*D* and *SI Appendix*, Fig. S8*A*). Two genes that are highly polymorphic relative to their size, *atp4* and *atp8*, encode components of the stator stalk of ATP synthase (32). The two genes with the highest absolute numbers of variants, *mp27* and *mp36*, are highly divergent genes containing homology to the mitoribosomal genes *uS3m* and *uS11m* (35) (*SI Appendix*, Fig. S8*B*), with long N-terminal extensions. Unusually, these genes have more nonsynonymous than synonymous changes (*SI Appendix*, Fig. S8*A*), possibly as a result of positive selection. The *D. discoideum* uS3m protein is also striking because it appears to be split across two polypeptides, Mp26 and Mp27 (35). We suggest that the apparent fast evolution of these genes might be driven by sexual selection. We also note that the transcript of another, poorly annotated polymorphic gene, *mp05*, is specifically enriched in gametes (36), and so could potentially influence organelle transmission during sex.

Sex originated early in eukaryotic evolution before the last eukaryotic common ancestor, but most likely after the first mitochondriate common ancestor (37). Before nuclear control mechanisms evolved to limit lateral transfer of organelles during sex, inheritance of mitochondria in the earliest sexual eukaryotes must have been a result of competition between endosymbionts, and so sex would have been an opportunity for competitive (proto-)mitochondrial genomes to spread in the population and escape from low-quality host environments (38), perhaps even before the fixation of the proto-organelle in the ancestral host population. One hypothesis for the origin of sex is that selfish elements first promoted cell–cell fusion as an efficient way to spread in this way (39), and it is possible that proto-mitochondria could have been the original selfish instigators of sex. Our findings also suggest that sexual selection could be a potent force promoting the retention of certain genes within mitochondrial genomes.

We did not find evidence that the reconfiguration of meiotic recombination in social amoebae is connected to the cannibalistic transfer of resources during macrocyst formation. Our data suggest that crossover designation is nonrandom, but the unconventional mechanism that operates in social amoebae remains mysterious; we have no strong candidate inducer of DNA breaks and cannot rule out the possibility that spontaneous lesions are used to initiate recombination (22). We suggest that the reconfiguration of chiasma formation in social amoebae, whether relying on spontaneous lesions or on a novel or co-opted active inducer of recombination, may have resulted from selection to increase recombination rates, perhaps to help neutralize hypothetical selfish drive elements (40) that might otherwise disrupt this cannibalistic system.

Our findings on mitochondrial inheritance raise questions about how the strikingly non-Mendelian character of *Dictyostelium* sex might affect the conflicts engendered by the dramatically different fates of cells during macrocyst formation. We predict further that important cell-biological consequences might follow from the mixing of many cells’ cytoplasms during sex, as it risks the spread of harmful endosymbionts, such as selfish mitochondrial genomes, viruses, and the bacteria present in certain isolates (41, 42). To the extent that outbreeding occurs in social amoebae, we expect that defenses against cytoplasmic parasites must arise concomitantly (43).

## Material and Methods

*Dictyostelium* amoebae were cultivated on bacteria, using standard techniques. The strains used in this study are described in *SI Appendix*, Table S1. Macrocysts were prepared by growth of mixed strains on LP agar plates submerged under buffer in the dark. The procedure for germination of macrocysts followed that of Wallace and Raper (25). A full description of these methods can be found in the *SI Appendix*, *SI Materials and Methods*. Care was taken to avoid the possibility of parental amoebae and spores being carried through to the germination plates; these can presumably become trapped among the fibrous material associated with macrocysts and, if bacteria from the growth plates are also present, can grow to greater numbers than the macrocyst progeny. Germination plates with unusually large numbers of fruiting bodies were invariably dominated by parental genotypes and were discarded. Details of methods for genome sequencing, read alignment and variant calling, identification of crossover sites, enumeration of mitochondrial and ribosomal DNA variants, PCR screening of mitotypes, generation of fluorescent protein reporter strains, and flow cytometry and fluorescence-activated cell sorting can also be found in the *SI Appendix*, *SI Materials and Methods*.

Whole-genome sequencing data and consensus parental genomes are available under the accession PRJEB28008 (44).

## Supplementary Material

Supplementary File
