# Supplementary material for: Triparental inheritance in Dictyostelium
Source: Proc Natl Acad Sci U S A. 2019 Jan 22;116(6):2187–92. doi: 10.1073/pnas.1814425116 (PMC6369745; doi:10.1073/pnas.1814425116)
Supplement: Supplementary File [file pnas.1814425116.sapp.pdf]

PNAS
www.pnas.org

Supplementary Information for

Triparental inheritance in *Dictyostelium*

Gareth Bloomfield, Peggy Paschke, Marina Okamoto, Tim J. Stevens, Hideko Urushihara

Correspondence should be addressed to Gareth Bloomfield
Email: garethb@mrc-lmb.cam.ac.uk

This PDF file includes:

SI Materials and Methods
Figs. S1 to S8
Tables S1 to S8
References for SI reference citations

www.pnas.org/cgi/doi/10.1073/pnas.1814425116

SI Materials and Methods

Growth of *Dictyostelium* cells, and cell fusion

Dictyostelium cells were grown in association with *Klebsiella pneumoniae* on SM agar plates (Formedium, Hunstanton, UK). Fusion competent cells were prepared by growth in shaken suspension (180 rpm) in heat-killed *K. pneumoniae* (OD_{600} of 10) in MSS buffer (5 mM MES, 10 mM NaCl, 10 mM KCl, 10 mM $CaCl_2$, pH 6.0) to a density of 3-6 x 10^6 cells per ml in the dark at 22 ºC, then incubated on ice for one hour, then cleared of bacteria by differential centrifugation (3 minutes, 300 *g*, 4 ºC) and washing in ice-cold MSS, repeated three times, resuspending the cells at a final density of 5 x 10^6 cells per ml in ice-cold MSS. TopA-GFP cells were grown in the presence of 10 µg/ml doxycycline to induce expression of the reporter. Cell strains were then mixed and shaken at 180 rpm and 22 ºC in conical flasks to initiate fusion. Cells were imaged in Lab-tek II chamber slides (Nunc) using laser-scanning confocal microscopes (Zeiss).

Macrocyst formation and germination

The three wild type pairwise crosses (two-way crosses) were between HM597 (a clone made in our laboratory of the David Francis stock of $A_2cyc^{r;}$ mating type II), HM598 (a clone made in our laboratory of the David Francis stock of of WS205; mating type I), and WS2162 (obtained from the Dicty Stock Center (1), mating type III). A_2cyc^r and WS205 were previously found to germinate at adequate frequencies, and to lack obvious chromosomal rearrangements (2) (3) (4). Macrocysts were produced by spreading 10^4 spores of each strain to be crossed along with stationary-phase *K. pneumoniae* on LP agar plates (1 g/L peptone, 1 g/L lactose, 12 g/L agar), overlaying the cells with 10 ml MSS buffer, followed by incubation in the dark at 22 ºC. In the three-way cross, 10^3 spores of HM597 and HM598 were mixed with 1.8x10^4 spores of HM1524, a *matA* deletion mutant of the AX2 strain. After 5-6 weeks, macrocysts were germinated according to the method of Wallace and Raper (2). Briefly, macrocysts were washed from the surface of the agar plate into centrifuge tubes then centrifuged and washed three times in MSS buffer (3 minutes, 300 *g*), resuspended in 10 ml 0.005% SDS and shaken for 210 minutes at 180 rpm at 22 ºC. The macrocysts were then washed twice in MSS before being resuspended in 10 ml 25 mM EDTA (pH 7.4) and shaken for 150 minutes as above. They were then washed twice in 0.025% dihydrostreptomycin solution then plated on 0.025% dihydrostreptomycin agar plates and incubated in ambient light at room temperature. Germination occurred

10-20 days later, at low frequency (<1%) and fruiting bodies containing progeny typically emerged from within, or lay adjacent to, ruptured macrocyst walls.

Spores from fruiting bodies likely to have formed from the progeny of single macrocysts were picked and cloned on SM agar plates. Putative progeny were initially checked for their *cycA* genotype by growth of SM agar plates containing 500 µg/ml cycloheximide, and screened by PCR with primers specific to *matA, matC,* and *matS* to test mating type. Progeny were also scored for SM agar plaque phenotype (HM597 has a sparse fruiting phenotype whereas HM597 and WS2162 are more densely fruiting). Recombinant progeny were obtained in multiple independent experiments.

Whole genome sequencing, read mapping and variant calling

Total genomic DNA was prepared using Zymo midi X kits and used to prepare libraries using the TruSeq, TruSeq No-PCR, or Nextera Mate-Pair protocols (Illumina), followed by sequencing on Illumina HiSeq and MiSeq systems. Progeny clones were sequenced to a median depth of 25x to 149x. Genome sequences for each parent (HM597, HM598, WS2162, and AX2; HM1524 is a clonally derived mutant of AX2 and so expected to bear a haplotype near-identical to AX2) were prepared by first aligning sequence reads from each strain onto the AX4 reference sequence (5) (6) using BWA-MEM (7) and variant calling using Freebayes (8), followed by iterative consensus calling using vcftools (9), and remapping of reads. For strains HM598 and WS2162, additional sequence reads generously provided by Jason Wolf were included in the mapping-consensus procedure. Reads from each progeny clone (XGB1, XGB2, XGB4, XGB7, XGB8, XGB57, and XGB58) were aligned and variants called against the sequence of the parent not used in that particular cross; potential PCR duplicates were removed before variant-calling. One copy of the chromosome 2 duplication present in AX4 was masked. Variants were filtered by rejecting positions with a read depth of less than four and/or a variant quality score less than 100. The cycloheximide-resistant parent strain HM597 has a C>T (proline to serine) variant at position 160 of the *rpl36a* gene, which causes a P53S change in ribosomal protein eL42. All of the cycloheximide-resistant progeny obtained from crosses involving this strain inherited this variant, while all cycloheximide-sensitive strains retain the wildtype allele. Variants at the same amino acid residue in the orthologous protein distinguish cycloheximide-sensitive and -resistant yeast species (10).

Identification of crossover sites

A program, Hycco, was written that uses Hidden Markov Models (HMM) to identify crossovers in haploid meiotic progeny (https://github.com/tjs23/hycco) using single nucleotide polymorphisms (SNPs) listed in pairs of VCF files (9) derived from the three genotypes (progeny clone and two parents). Hycco works by first segmenting each chromosome into binned regions of a specified length (here we used 10 kb). Within these regions the presence of SNPs, that can distinguish one parental genotype from the other, is input to train a Gaussian HMM using the Baum-Welch method. This gives maximum likelihood estimate of the probabilistic HMM parameters using only the observable SNP data.

The HMM is then interrogated for the probabilities of its underlying (hidden) parental genotype states (labelled as A or B) using the Forward-Backward method. This assigns one of the two parental genotype states (A or B) to contiguous segments of the chromosomes. Where the genotype state of highest probability swaps between A and B a chromosomal crossover is inferred. The crossover point is then more precisely estimated, at a resolution better than the initial HMM segments. Here the closest pair of parent-specific SNPs to the HMM segment edge that swap from A to B or from B to A, in the same way as the HMM state change, are sought. The crossover point is then estimated as halfway between these two A/B distinguishing SNPs. Crossover positions were confirmed by manually identifying the flanking variants.

Enumeration of mitochondrial and ribosomal DNA variants

After mapping total genomic reads to the reference genome used in each cross and variant calling, we estimated relative proportions of each mitotype in each clone by extracting all reads mapped to the mitochondrial genome and counting reads matching k-mers exactly matching specific variants plus ten base pairs to either size. K-mers were counted around positions 8133, 14151, and 44620 on the mitochondrial genome, and 15748, 16036, and 27347 on the ribosomal DNA palindrome (coordinates in the AX4 reference genome, as available from http://dictybase.org/db/cgi-bin/dictyBase/download/blast_databases.pl, dated 05-13-2009).

To assess broader patterns of variation among *D. discoideum* mitochondrial genomes, we analysed the genomes of twenty additional isolates (11) using the same mapping and variant-calling procedure described above, and enumerated synonymous and non-synonymous variants in mitochondrial protein-coding sequence in them, along with the four parental strains used in our crosses. All mitotypes were >99.5% identical in sequence overall, with the exception of that of NC105.1, which contains three apparent deletions >500bp relative to all others (one corresponding to the final group I intron of the *cox1/2* gene, one to the group I intron of the *rnlA* gene, and another affecting two short unannotated ORFs immediately downstream of *cox1/2*), as well as >100 substitution variants unique among known mitotypes. The numbers of non-synonymous variants per kilobase across this dataset were colour-coded on a circular representation of the protein coding genes of the mitochondrial genome using the 'circlize' package in R (12).

Chromosomal intervals co-segregating with rDNA genotype were determined in R using data from all seven progeny for which whole sequence data are available. We also attempted to find mate pair reads spanning any gaps between a rDNA chromosomal copy and contigs present in the genome assembly, but have not identified any convincing candidates.

Mitotype screening by PCR

The HM1524 mitotype differs from the other by an 8 bp insertion relative to the other parental strains at position 6956. The two oligonucleotide primers 5'-CGTTTCTCTAAATTACATATATATATATATATATATACTG-3' (HM1524) and 5'-CCGTTTCTCTAAATTACATATATATATATACTG-3' (other strains) were used in separate PCRs with the common primer 5'-AGATTTTGGTGAAAAAATGGAGTTA-3', using the cycle parameters 96 ºC for 30 s, then 25 cycles of 10 s 96 ºC, 15 s 59 ºC, 60 s 68 ºC, then 300 s 68 ºC. For progeny without whole genome sequence data support, amplicons from this PCR were Sanger-sequenced and the specific match to the HM1524 at position 6175, at which the other parent strains differ, was confirmed. Dominant mitotype was checked by PCR using the primers 5'-GATGCTGTTAAAGCTTTCAATCCA-3' and 5'-ACTAGAAAGTTTACCTTACTAGAG-3' and Sanger sequencing to check positions 16801 and 17193.

Fluorescent protein reporters and transformation

Histone 2B was tagged at the C-terminus with GFP, cloned into a pDM vector containing a *hygR* gene driven by the *act14* promoter (13). The first 258 bp of the *topA* gene was cloned to the 5' of the *GFP* gene in pDM340, which contains *neoR* driven by the *act8* promoter (14). A vector containing the mitochondrial targeting motif of GemA tagged to the C-terminus of tagRFP and *hygR* driven by the *act8* promoter (15) was a gift from Jason King. To image fusion of *matA* null cells, strain HM1524 was transformed with pDXA-GFP (16), HM1558 (a type III strain whose derivation will be described fully elsewhere) was transformed with pDXA-mRFPmars (17). Cells were transformed by electroporation (18) and selected in diluted SM broth with antibiotic-killed *K. pneumoniae* (19) in the case of strain HM598, or axenically in HL5 medium (AX2 and HM1558) in 100 µg/ml hygromycin or 20 µg/ml G418.

Flow cytometry and fluorescence-activated cell sorting

H2B-GFP and GemA-tagRFP cells were mixed in a ratio of 50:50 and co-developed on KK2 agar plates (16.5 mM KH_2PO_4, 3.9 mM K_2HPO_4, 2 mM $MgSO_4$, 0.1 mM $CaCl_2$, 12 g/L agar). As controls non-mixed cells were used. After at least 24 h of development the ripe spore heads were harvested with an inoculation loop and transferred into KK2 buffer. To remove the sorocarp and avoid clumping of spores the suspension was filtered using a 10 µm filter (Symex) to filter the sample. The filtrate was transferred into conical centrifuge tubes at low density (about $2x10^5$ spores per ml) and used either for cell sorting or flow cytometry. For flow cytometry the samples were run on a LSR II flow cytometer at low speed to avoid doublet events. At least 50000 spores were analysed per sample. To filter for GFP fluorescence a B525 filter and for tagRFP a YG610 filter was used.

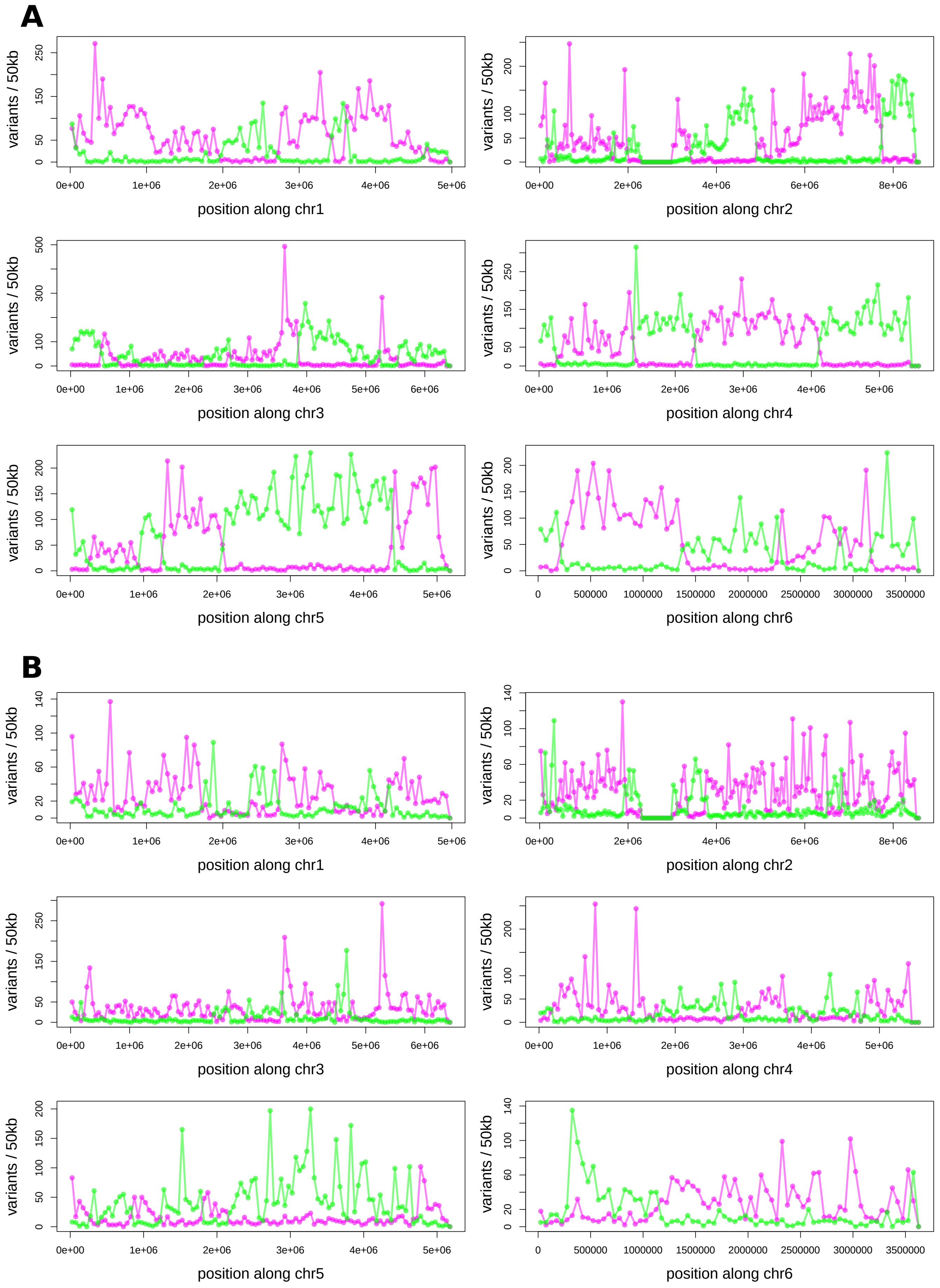
A
B
variants / 50kb
position along chr1
position along chr2
position along chr3
position along chr4
position along chr5
position along chr6

Figure S1. Recombination in haploid progeny clones XGB57 and XGB58. A. The number of variants specific to each of strain XGB57's parents, HM597 (plotted in magenta) and WS2162 (green), were tallied in 50 kb bins along each chromosome. **B.** Variants were counted and plotted as above for strain XGB58: the parents of this clone are HM598 (magenta) and WS2162 (green).

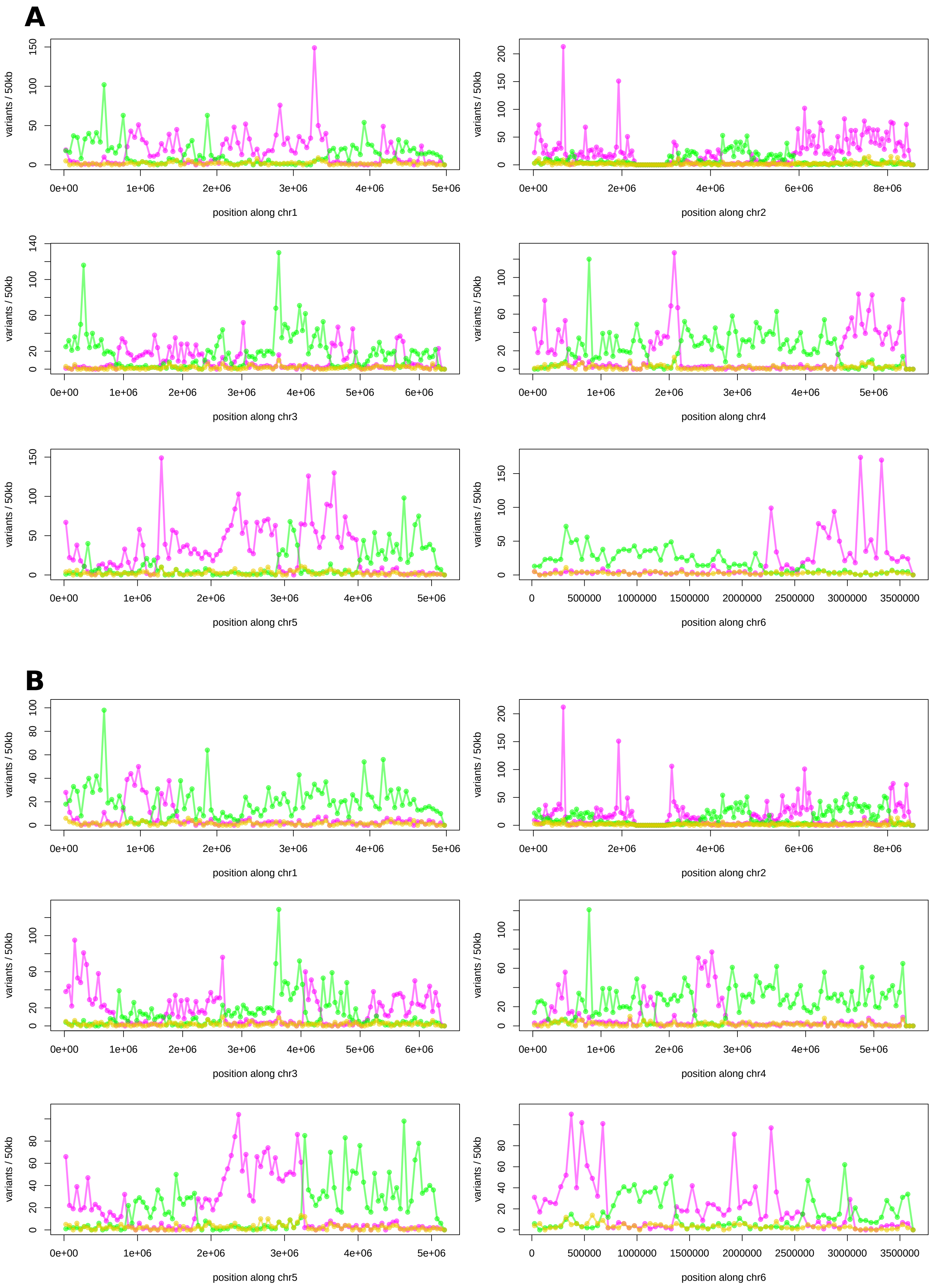
A
variants / 50kb
position along chr1
position along chr2
position along chr3
position along chr4
position along chr5
position along chr6
B
variants / 50kb
position along chr1
position along chr2
position along chr3
position along chr4
position along chr5
position along chr6

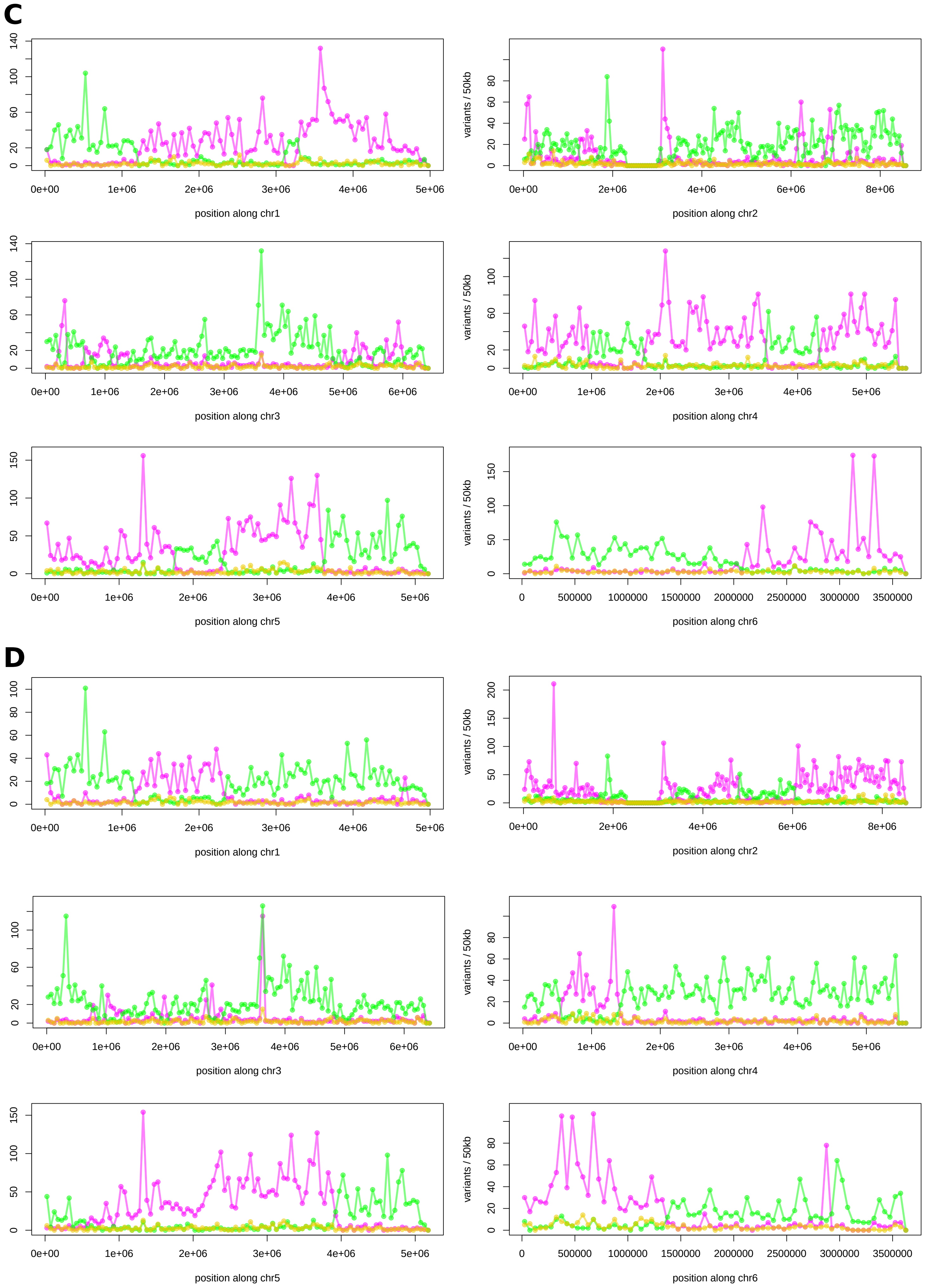
C
variants / 50kb
position along chr1
position along chr2
position along chr3
position along chr4
position along chr5
position along chr6
D
variants / 50kb
position along chr1
position along chr2
position along chr3
position along chr4
position along chr5
position along chr6

Figure S2. Recombination in haploid progeny clones from three-way crosses. The number of variants specific to each of the parents of strains XGB2 (**A**), XGB4 (**B**), XGB7 (**C**), and XGB8 (**D**) were tallied in 50 kb bins along each chromosome and plotted. Variants specific to parent strain HM597 are shown in magenta, those specific to HM598 in green, and HM1524 (AX2 *matA* null) in gold.

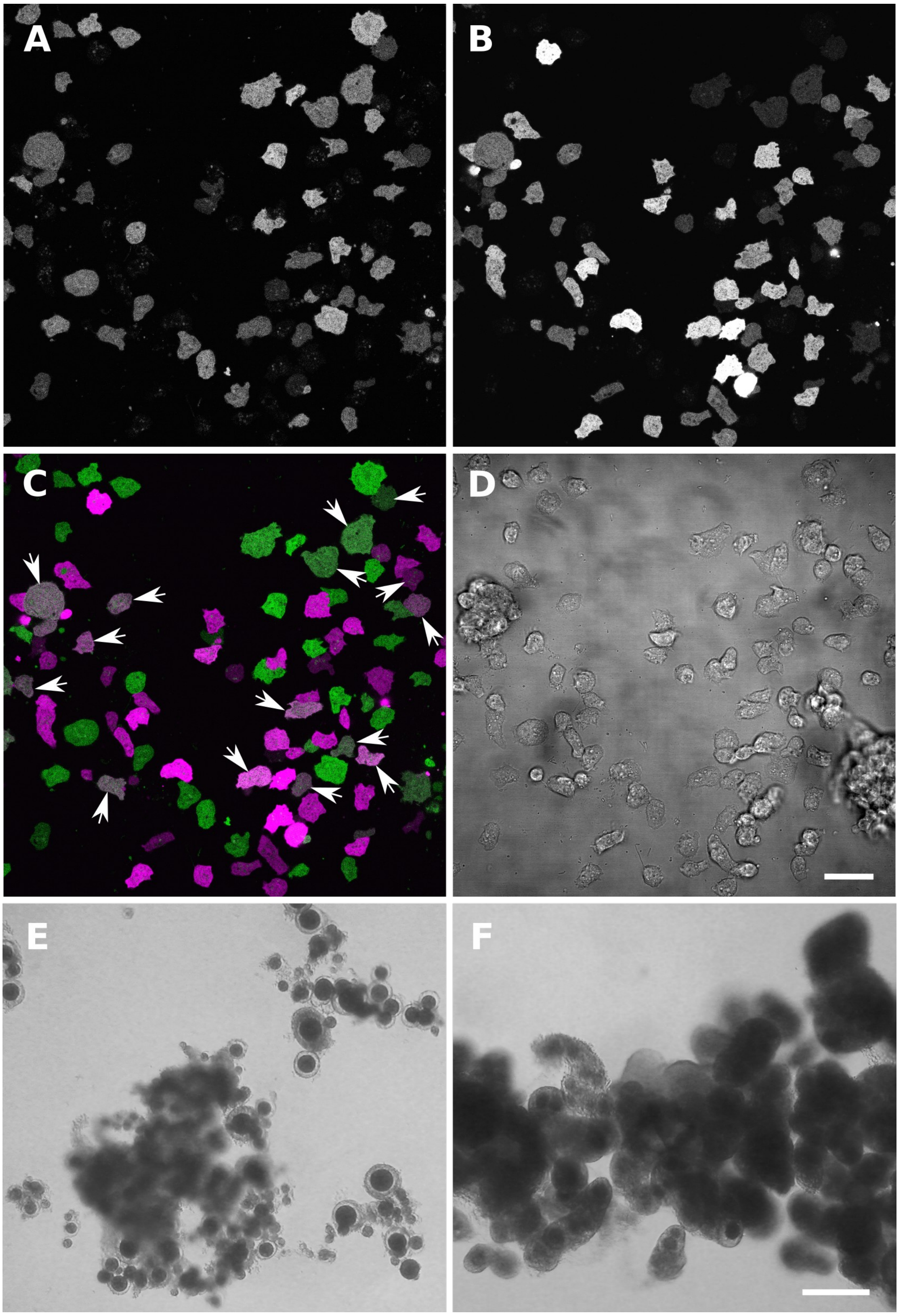

Figure S3. HM1524 (*matA* null mutant) is able to fuse but is unable to sexually differentiate correctly. GFP-labelled HM1524 (**A**) and RFP-labelled HM1558 (type III, **B**) were grown in fusion-competent conditions then mixed and imaged after one hour. Fused cells containing both GFP and RFP are marked in **C** with arrows. **D** shows the same cells in digital interference contrast. The extent of fusion is highly variable between different experiments, likely explaining why we have only obtained majority laterally-transmitted mitotypes so far in a small number of progeny from the same experiment. Scale is 25 µm. **E**. Wildtype macrocysts formed in a cross between strains NC4 and V12M2, after

incubation on LP agar plates in association with *Klebsiella pneumoniae*, submerged under MSS buffer in the dark. **F**. HM1524 grown in the same conditions; these cyst-like structures are observed infrequently in this strain, but have not so far been observed in a strain (HM1525) that has the same *matA* deletion in a different genetic background. Scale in **E** and **F** is 250 μm.

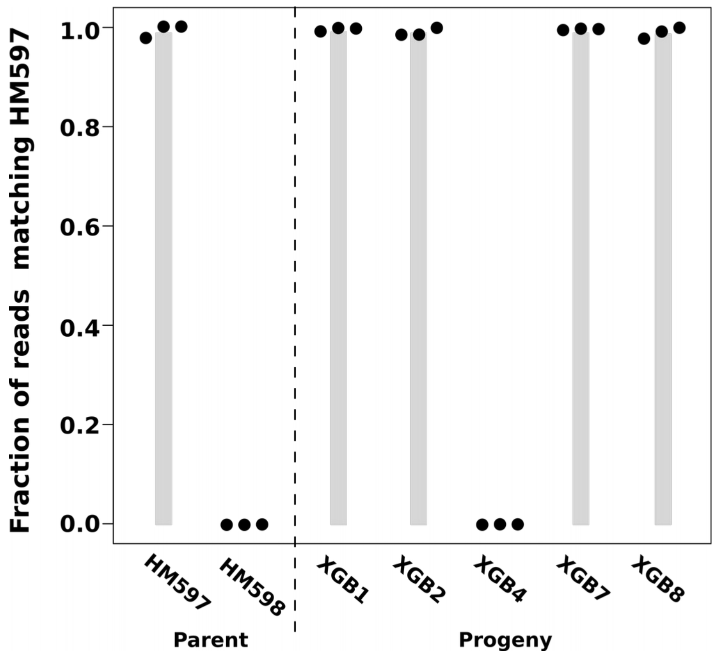

Figure S4. Ribosomal DNA inheritance in *D. discoideum* is largely uniparental. The fraction of ribosomal DNA reads in whole genome sequencing data matching strain HM597 was counted at three sites at which HM597 and HM598 differ. Sequence data for the rDNA palindrome are noisy because multiple fragments of the sequence, varying in sequence, appear to be present in the genome, so we cannot confidently assess whether inheritance of this sequence is truly uniparental with this dataset.

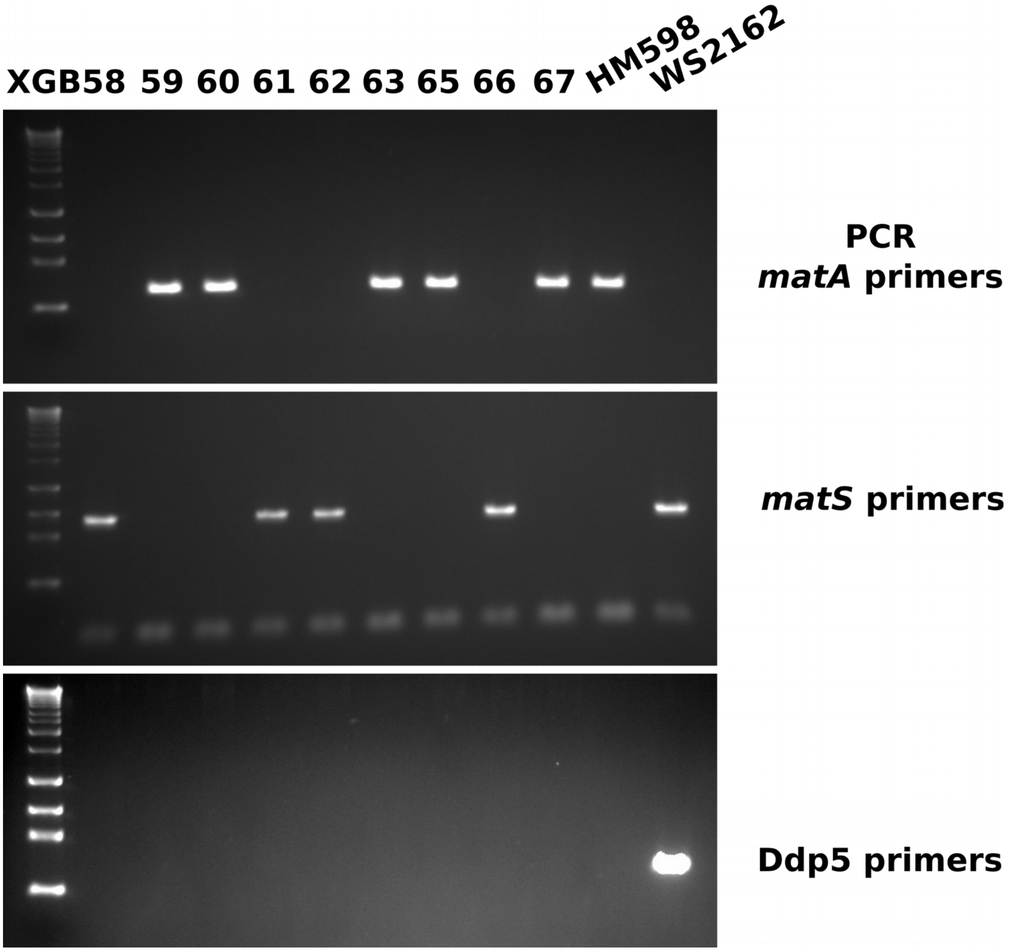

Figure S5. Nuclear plasmids are not inherited by *Dictyostelium* meiotic progeny. We found that whole genome sequencing data from the two progeny clones having WS2162 as one parent, XGB57 and XGB58, lacked any reads mapping to the nuclear plasmid Ddp5, which is carried by that parent. We extended this observation by screening for the presence of the plasmid in a further ten progeny clones from the HM597 x WS2162 cross (not shown), and eight from the HM598 x WS2162 cross (shown here). None of these progeny inherited the plasmid, as assessed by PCR. PCRs for *matA* (specific to mating type I) and *matS* (specific to mating type III) act as positive controls. These data could be explained by a genetic incompatibility between the plasmid and one or more genes present in the other parents, or by a mechanism that eliminates nuclear parasites specifically during.

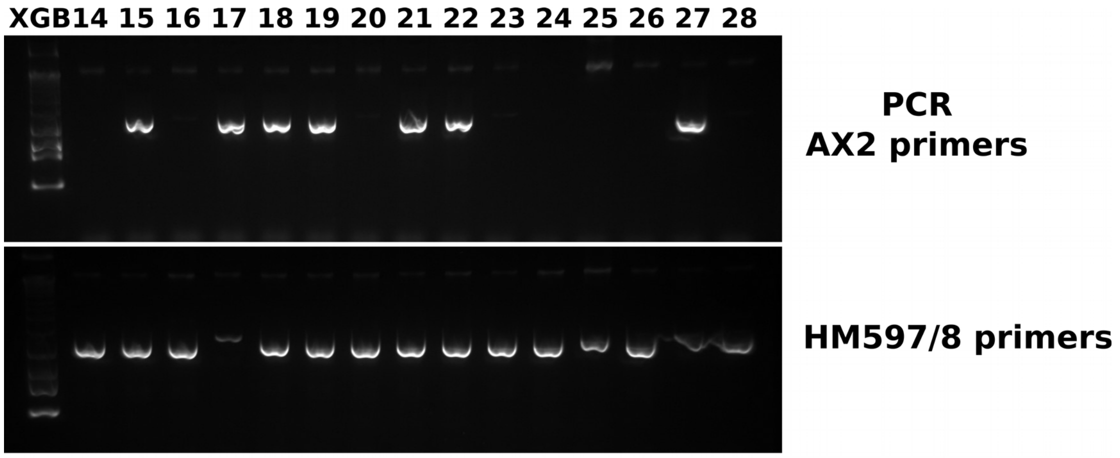

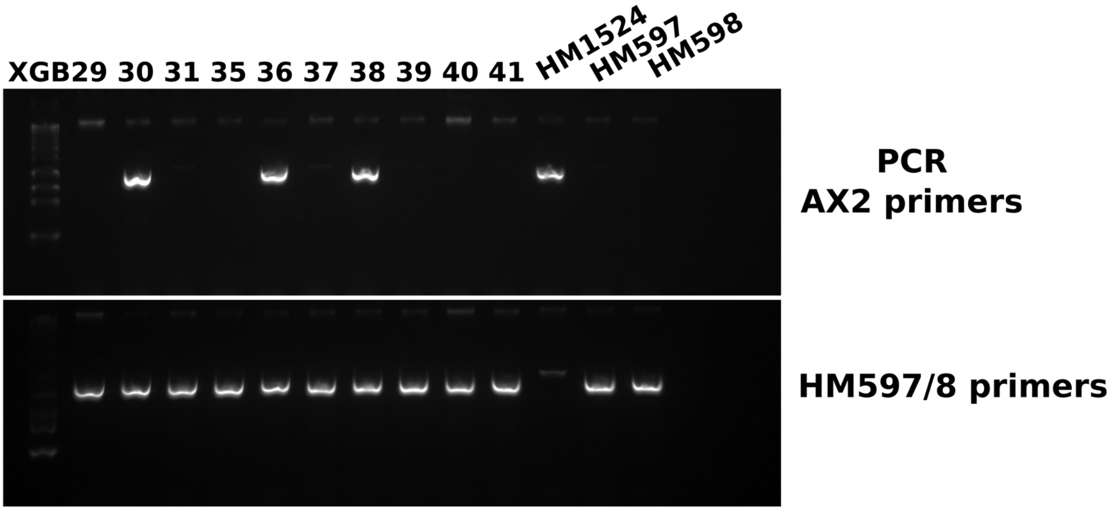

Figure S6. Lateral transmission of mitochondrial DNA into selected progeny clones. PCRs were performed with mitotype-specific primers that span one variant in the mitochondrial genome that distinguishes the AX2 mitotype (borne by the *matA* null strain) from the two wildtype strains used in the three-way cross, HM597 and HM598. Most of these progeny carry the HM597/8 variant, and Several also contain the AX2 variant. One progeny clone, XGB17, was the only example that lacked the HM597/8 variant here; however it possesses predominantly the HM598 mitotype at a location approximately nine kilobases away, as detected by Sanger sequencing of a PCR amplicon (see table S1), suggesting that recombination between these two mitotypes has occurred.

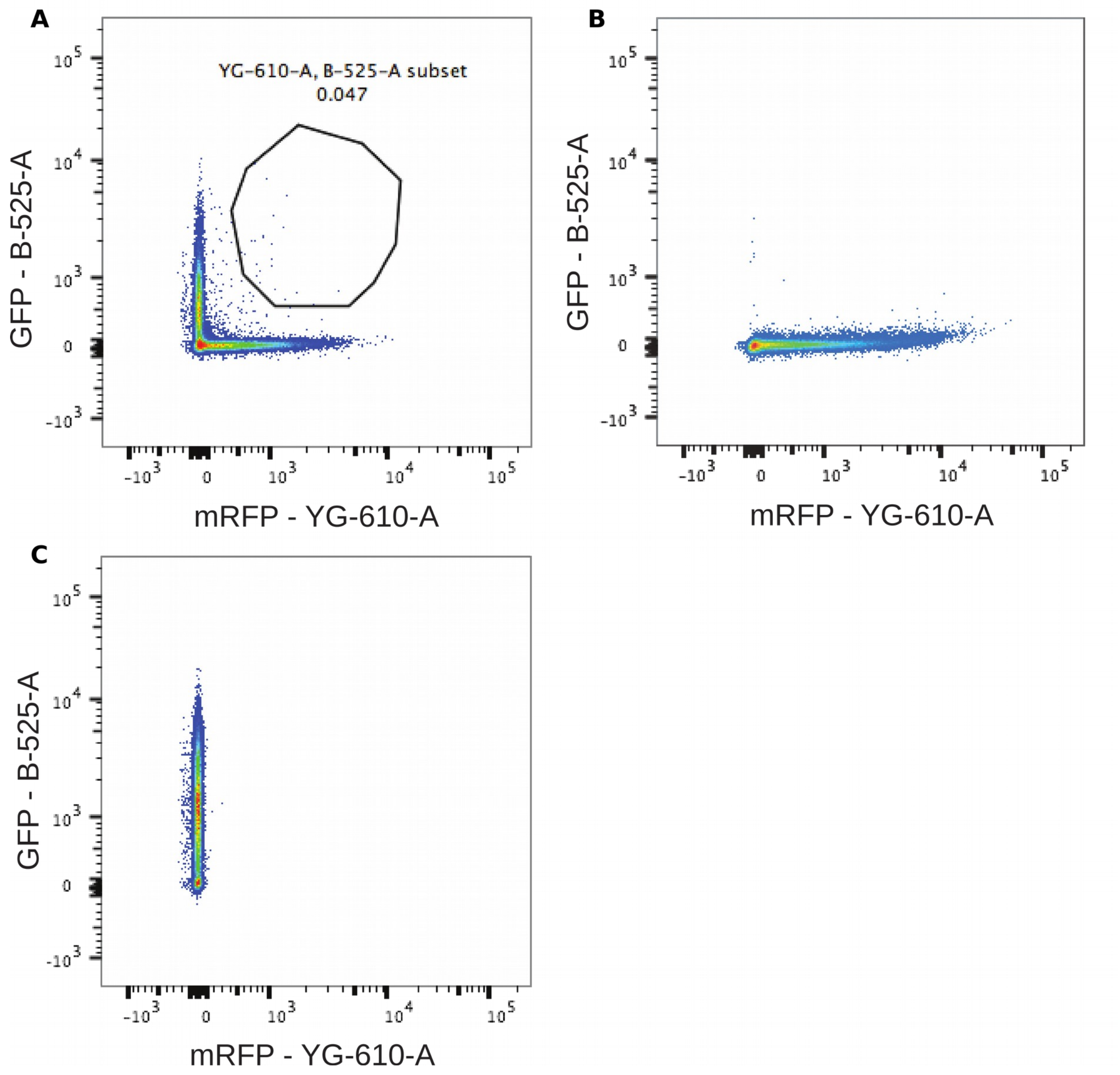

Figure S7. Lateral transfer of mitochondria during asexual development in *Dictyostelium*. Haploid AX2 cells containing either histone-GFP or mitochondrial-RFP reporters were mixed and co-developed to produce asexual fruiting bodies. In this asexual process, social amoeba cells remain separate, and differentiate into stalk and spore cells. Stalk cells undergo a form of programmed cell death as they lay down cell walls; spore cells, carried at the top of the stalk, can survive. **A.** By harvesting spores, and measuring their fluorescence by flow cytometry, we found that approximately 10^{-4} to 10^{-3} cells contain both GFP and RFP reporters, indicating a lateral transmission event: mitochondria and nuclei that started out in different cells are now united in the same cell. This could be due to whole-cell fusion resulting in diploid cells (parasexual fusion, known to occur at lower frequencies than this), or transient fusions result in transfer of mitochondria. **B** and **C** show controls for RFP- and GFP-only conditions. A single representative experiment is shown; in this case the fraction of fluorescent cells in the mix experiment carrying

both reporters is 0.047, roughly two orders of magnitude higher than a typical parasexual fusion frequency.

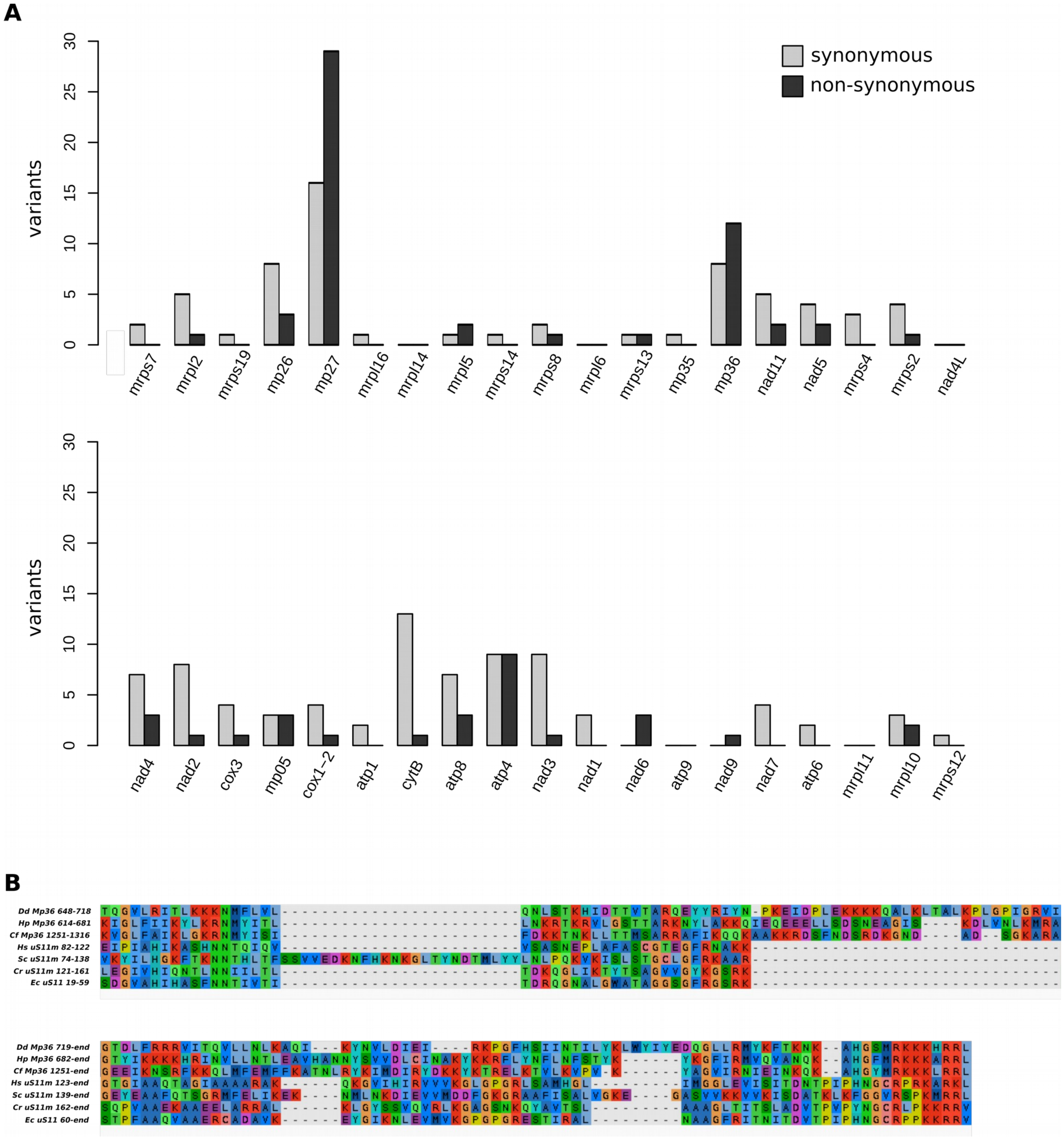

Figure S8. Polymorphisms in the *D. discoideum* mitochondrial genome. A. Synonymous and non-synonymous variants within the coding sequences of mitochondrial genes in 24 isolates were enumerated; homing endonuclease genes were omitted from the analysis. Compound variants were counted according to the number of separate codons affected. The possession of more non-synonymous than synonymous variants is often considered to indicate the effect of positive selection. **B.** The C-terminus of *D. discoideum* (*Dd*) Mp36 is aligned with orthologues from *Heterostelium pallidum* (*Hp*) and *Cavenderia fasciculata* (Cf), along with uS11m from *Homo sapiens* (Hs), *Saccharomyces cerevisiae* (Sc), and *Chlamydomonas reinhardtii* (Cr), and uS11 from *Escherichia coli* (Ec). Descent of *mp36* from the ancestral *uS11m* gene is supported by its

position on the mitochondrial genome. The adjacent gene, *mp35,* was previously suggested to be the *uS11m* homologue for the same reason (35); however Mp35 is a predicted transmembrane protein and we cannot detect any sequence homology with uS11m.

Table S1

Strain	Parent	Mating type	*cycA*	Predominant mitotype	AX2 mitotype present
HM597	A_2cyc^r	II	-	597	
HM598	WS205	I	+	598	
WS2162	-	III	+	2162	
HM1524	AX2	null	+	AX2	+
XGB1	HM597 x HM598	II	-	597	
XGB2	HM597 x HM598 x HM1524	II	+	AX2	+
XGB4	HM597 x HM598 x HM1524	II	+	597	-
XGB5	HM597 x HM598	II	-	597	
XGB7	HM597 x HM598 x HM1524	II	-	597/AX2	+
XGB8	HM597 x HM598 x HM1524	II	+	AX2	+
XGB9	HM597 x HM598	I	+	597	
XGB10	HM597 x HM598	I	+	597	
XGB11	HM597 x HM598 x HM1524	I	+	597	-
XGB12	HM597 x HM598 x HM1524	I	+	597	-
XGB13	HM597 x HM598 x HM1524	I	+	597	-
XGB14	HM597 x HM598 x HM1524	II	-	597	-
XGB15	HM597 x HM598 x HM1524	I	-	598	+
XGB16	HM597 x HM598 x HM1524	II	+	597	-
XGB17	HM597 x HM598 x HM1524	I	+	598†	+
XGB18	HM597 x HM598 x HM1524	I	+	597/598 equally	+
XGB19	HM597 x HM598 x HM1524	II	-	597/598 equally	+
XGB20	HM597 x HM598 x HM1524	I	-	597	-
XGB21	HM597 x HM598 x HM1524	I	+	597	+
XGB22	HM597 x HM598 x HM1524	I	-	597	+
XGB23	HM597 x HM598 x HM1524	II	+	597	-
XGB24	HM597 x HM598 x HM1524	I	-	597	-
XGB25	HM597 x HM598 x HM1524	I	+	597	-
XGB26	HM597 x HM598 x HM1524	II	-	597	-
XGB27	HM597 x HM598 x HM1524	II	+	598	+
XGB28	HM597 x HM598 x HM1524	II	+	597	-
XGB29	HM597 x HM598 x HM1524	I	+	597	-
XGB30	HM597 x HM598 x HM1524	II	-	598	+
XGB31	HM597 x HM598 x HM1524	II	+	597	-

**Strain**	**Parent**	**Mating type**	***cycA***	**Predominant mitotype**	**AX2 mitotype present**
XGB32	HM597 x HM598 x HM1524	II	+	597	-
XGB33	HM597 x HM598 x HM1524	II	-	597	-
XGB34	HM597 x HM598 x HM1524	II	-	597	-
XGB35	HM597 x HM598 x HM1524	I	+	597	-
XGB36	HM597 x HM598 x HM1524	II	-	598	+
XGB37	HM597 x HM598 x HM1524	I	+	597	-
XGB38	HM597 x HM598 x HM1524	I	-	597	+
XGB39	HM597 x HM598 x HM1524	II	-	597	-
XGB40	HM597 x HM598 x HM1524	II	+	597	-
XGB41	HM597 x HM598 x HM1524	II	+	597	-
XGB42	HM597 x HM598 x HM1524	I	+	597	-
XGB43	HM597 x HM598 x HM1524	II	-	597	-
XGB44	HM597 x HM598 x HM1524	I	+	597	-
XGB45	HM597 x HM598 x HM1524	II	+	597	-
XGB46	HM597 x HM598 x HM1524	II	-	597	-
XGB47	HM597 x HM598 x HM1524	II	-	597	-
XGB48	HM597 x HM598	I	+	597	
XGB49	HM597 x HM598	II	-	597/598 equally	
XGB50	HM597 x HM598	II	-	597	
XGB51	HM597 x HM598	I	-	597	
XGB52	HM597 x HM598	I	+	597	
XGB53	HM597 x WS2162	II	+	n.d.	
XGB54	HM597 x WS2162	II	-	n.d.	
XGB55	HM597 x WS2162	II	+	n.d.	
XGB56	HM597 x WS2162	II	+	n.d.	
XGB57	HM597 x WS2162	III	-	597	
XGB58	HM598 x WS2162	III	+	2162	
XGB59	HM598 x WS2162	I	+	n.d.	
XGB60	HM598 x WS2162	I	+	n.d.	
XGB61	HM598 x WS2162	III	+	n.d.	
XGB62	HM598 x WS2162	III	+	n.d.	
XGB63	HM598 x WS2162	I	+	n.d.	
XGB64*	HM597 x HM598	I	+	597	
XGB65	HM598 x WS2162	I	+	n. d.	
XGB66	HM598 x WS2162	III	+	n. d.	

Strain	Parent	Mating type	*cycA*	Predominant mitotype	AX2 mitotype present
XGB67	HM598 x WS2162	I	+	n. d.	
XGB68*	HM597 x HM598	I	+	597	
XGB69	HM597 x WS2162	III	+	n. d.	
XGB70	HM597 x WS2162	III	-	n. d.	
XGB71	HM597 x WS2162	II	+	n. d.	
XGB72	HM597 x WS2162	II	+	n. d.	
XGB73	HM597 x WS2162	III	+	n. d.	

Table S1. Parental and progeny strains analysed in this study. "Predominant mitotype" is defined as making up >90% of the total mtDNA sequence as assessed either by whole genome sequencing of Sanger sequencing of PCR amplicons. "*cycA*" refers to cycloheximide resistance/sensitivity: "+" refers to the wildtype allele (cycloheximide sensitive), "-" is the mutant allele (cycloheximide resistant). "N. d" means "not determined". Grey boxes in the final column indicate that the strain in question is not the result of a cross involving HM1524, and so in most cases the presence of the AX2 mitotype was not tested. (*) Multiple clones from the macrocysts giving rise to progeny XGB64 and XGB68 were genotyped/phenotyped. For XGB64, all 21 clones were identical; for XGB68 all ten clones were identical. In all other cases where we tested multiple clones from a macrocysts (not recorded in this table), all clones have been found to be identical. (†) PCR evidence suggests that recombination may have occurred between the AX2 and HM598 mitotypes in XGB17 (see Fig S3).

Table S2

Strain	Chromosome	Sequence	Location	Fraction (G+C)
XGB58	3	AAAAAAAAAAATAATATACAAAACTAATTAATGATGATCAAAAACAAGTTGAATGATTGGTA	Intergenic between DDB_G0279673 and DDB_G0295731	0.180
XGB57	4	CATAAAATAAAGATCATTTGAATTTTATTTTATTTTTTGATGATCTACCCTCCTTATTTTTTC	Intergenic between DDB_G0283117 and DDB_G0283119	0.206
XGB1	6	AAAAAAAAAAAAAAAAAAAAAATGAAAAAAACCCTTTTATTTTTTGAATTTTTTTTTTTTTGAATTTTTTTTTTTACACAAAAATT	Intergenic between DDB_G0292426 and DDB_G0292524	0.095
XGB2	2	TTATAATTATTATTATTATTAAAAATAAAAATTTTTTAAAACTTTTTTTTTTTTTTTTTTTTTTTATTACAAATGAAATCAATTCAAAAAT	Intergenic and 5' end of DDB_G0271802	0.055
XGB4	3	AACCATCATTATCATCATCATTATCATTTTCTTGATTTTCAATCTCTTTTCTTTTATTATTATTAACCATCTTTTTTTTTTTTAAACTGTAAAAAAGTGTGCAGGAGCAAAAAAAAAGAAATTTTTTTTTTTTTTTTTTTTTTTTTTAAAATTAAAAAATAAAAAAGGC	Intergenic and 5' end of DDB_G0281305	0.180
XGB58	3	AAAAATTACAATTGTAATTTTTTAAATATTTTATAAATTTAAATAATATTAATAAAATGAATAATTACTCTTTTCTAGTTTGCTCATCAGATAATACTACATATGCTTCAGAAATGATTTTAAATATGTCTGCAGCGTCTTTTTTTAAGACAATCAAAAAGATGTTGC	Within DDB_G0280741	0.202
XGB2	5	TTTTTTTTTTTTTTTTTAAGAATAATTAAGGTGGAAAATAATAAAAATAATATTAAAAAAAAAAAATTAAAAAAAAAATGTTTGGATTAATTCAATGGAAATGAAAAGAAGATAGGTTTGTCAAAATTAGGTTTTAAAATAAGTAAAGCTGCAAATATAAAAAAAAAACAAATATTA	Within DDB_G0289537	0.149
XGB57	5	ATTATTATCATACAAGTAGTAAACAAAGGAAGAGTATTTGATGTTTATCAAGTATGTGTTTATTTTATTTTATTTTATTTTTTTGTAAAATTTTTATTTTTCACT	Intergenic between DDB_G0290613 and	0.198

		CGATTAAATTGAAACTAATTTCTTTT TGATCCTTTATTATTTACAAATATTCT ACTATCCATCATTTTCTCC	DDB_G0290615	
XGB58	2	AACCAATTTTTTTTTTTTTTTTTTTTT TTTTTTTTTACTTTTTTTTTTTTTACT TTTTTTTTTTACTTTTTTTTTTTTTTT TTTTTTTTTTACTTTTTTTTTATTTTT GCTCAAATTTGTTTTAATTCTCTTCT TTTGTTTTTTGTGTATTTATTTATTTA TTTATTTATTTATTTT	Intergenic between DDB_G0271332 and DDB_G0271362	0.090
XGB1 and XGB2	6	ACCATAGTCGAGTAAAAATTAAAAT ACTAAAAATAATAATAAATAATAAAA TGGAAATAAAAATTTATATTTCTTAT TTAAACCCATACCACCTGGCGAATA AAATCTAAAAAACAAATGGTTTGTA TGGGGTAAATTAAATAATAATTTTTA ATGAAAAAAATTCTTAAAATATGTA AAA	Intergenic between DDB_G0295421 and DDB_G0293698	0.182
XGB4	4	AAATAACTAAGAAAAAATGATAAAA ATTATAAAAATCAATCTCTTCTTTAC TTTAAAAGAAAAGCAAATTTACAAA ATTTTTCCTTTTTTCTAACAATTTTA AAAATACACAACTATTTAAACAAAA AGTTAACTTTTTCCCACTTTTTAAAG TTAGAAAATTACTTTTTTTTTTATTAT TATTATTTTTTAT	Intergenic between DDB_G0284103 and DDB_G0284105	0.150
XGB2	1	TTGTCAGCGATAGCGGTGTTATGTA GCTCGAGGGAGGATAGTAGAAAAC AAGTCGTAGATGCAAAGATAATACC ACAAATTATAAGTAGTTTAAATTCAA GCAATTACGCAATCAGAGCAGCAGC ATGTAATTGCACGAAATCATTATCAA GATCGATTAAACAATTGCGTACCTC ATTATTCGATTGTACGATTGCA	Within DDB_G0270626	0.369
XGB8	2	TTTTCTTCCTCTTCATCATCTTTTAAT TTTTTATTTGATTTTTTAGTGGCAGC TGGTGGTTTCTTTTTTGTTTCTTTTT CTGTTGATTTCTTTGGTGTTTCATCT TCTTCCTCTTCCTCTTCCTCTTCTTT CTTTTTAGATGATTTTGAATCTTTTTT AGGAGTTGATTTCTTCTTTGGTTTTT TCTCTTCCTCATCTTCTTCCTCCTCC TCCTCCTCCTCC	Within DDB_G0274195	0.338
XGB7	1	CCAAATATAAAGAAATTTCCATCTCC TATTACTAAATTTAAATTTTATATATT TTAATATACTTCTTTTTAAATATATTT TTTTTTTTATTTTTTATTGTTATTACCT	Intergenic between DDB_G0269674 and	0.122

		AATAATTCTAATTTTCAAAAAAAAAAAAAAAAAAAAAAAAACAAATTCAAAACCACTGTACCAATTATCTAAAAAATTATTTAAATTTATATTTTATTCAATATTTAAAAAAAAGTTTGTTTTAAAAGTTA	DDB_G0270606	
XGB7	2	TTTATTTTTTTATTTTTTTGATTTTTAATTATAATTATTGTTATATGAGTGTGTGTCTTTGATGTCAAAATGTATAAGAAAATTAATTTAAAAAATAAAAAAGAGTAAAAAAAAATAAAATAAAAAAAATAAAAATAAAATAAAATAATAAAAAATTTAATAAATTAAAATTTTAATATACCCTAGATATTTTCGTGTAGCATTTAAAAGTGCAATATTCTTTTTTTCAAATTCTTTTTTTTTTTTTTTC	Intergenic between DDB_G0272090 and DDB_G0272076	0.124
XGB1	4	TTTTTTTTTTTTTATAAAATTTATGTTCCAAAACTTACAAATTAAATAAATAGTATTTGAATAAATAAAACAAATATTTGTGTTGAGTGAAAAAAAAAAAAAAAAAAATGAAAAATGAAAAAAAAAAAAAAAAAAAAAATGAAAAAATCAACACAAATTTTCTATTTATATGAATATTCAATGAATGTAATATTACCAATATATTTCAAAAAAATTTTTTAATAATTTCAAAAAAAAAAATTTGAAAAAAAAATA	Intergenic between DDB_G0284903 and DDB_G0284905	0.115
XGB58	6	CTATTTCATGATGAGAGAATTCAAACATTAGTAGGTTTCCAAATTATTAATATAAAGAGTGATTTGAAATCATTGAGAAAAGAAATTGATGATATGGAAATTCCAGAATTAAAAGGTTTGGAATTTAGATTCATGTTAAAAAAAATTCCAATTTCTTTGAAACAAGAATCAAATTATATTATTAAAGATTGTTTATTCTCAATGAATGGTAGAATTACAATTGAAATTAGATCAATCATTCCAACAATTGTTTCTGC	Within DDB_G0293942	0.241
XGB7	5	GCCTCTTTAATCGAAAAGAGAGGTTCACGTTTCCTTGCTGAATGGGCTGCCGTTATGACTGGTGTCAAACCAAAGACTTGGTTCCTTCGTCCAGATGTTGGTCCATTAATTGTCTTAGAAATCTTTGGTGAATGTTTCAGAAGAGTTTTCCAAGGTAAAAAACAAATTAAAAAATTAGATTAAATTTCTCAAATTATAATTAAAAAATCAAAAAAACTATAGATCAATTTCAATATGTAATAATTTTTTTAAAAAAAAAAAC	Intergenic and 3' end of DDB_G0289157	0.294

Table S2. Locations, sequences, and (G+C) composition of crossover intervals less than 300 bp. Crossovers regions are defined in this study as the sequences between two genetic variants that are of different parental origin in a given progeny clone. (G+C) composition was calculated according to the reference genome against which reads were mapped in each case.

References for supplementary materials

1. Fey P, Dodson RJ, Basu S, Chisholm RL (2013) One stop shop for everything *Dictyostelium*: dictyBase and the Dicty Stock Center in 2012. *Dictyostelium Discoideum Protocols*, Methods in Molecular Biology. (Humana Press, Totowa, NJ), pp 59–92.

2. Wallace MA, Raper KB (1979) Genetic exchanges in the macrocysts of *Dictyostelium discoideum*. *Microbiology* 113(2):327–337.

3. Francis D (1998) High frequency recombination during the sexual cycle of *Dictyostelium discoideum*. *Genetics* 148(4):1829–1832.

4. Bloomfield G, Tanaka Y, Skelton J, Ivens A, Kay RR (2008) Widespread duplications in the genomes of laboratory stocks of *Dictyostelium discoideum*. *Genome Biol* 9:R75.

5. Eichinger L, et al. (2005) The genome of the social amoeba *Dictyostelium discoideum*. *Nature* 435(7038):43.

6. Basu S, Fey P, Jimenez-Morales D, Dodson RJ, Chisholm RL (2015) dictyBase 2015: Expanding data and annotations in a new software environment. *genesis* 53(8):523–534.

7. Li H (2013) Aligning sequence reads, clone sequences and assembly contigs with BWA-MEM. *ArXiv13033997 Q-Bio*. Available at: http://arxiv.org/abs/1303.3997.

8. Garrison E, Marth G (2012) Haplotype-based variant detection from short-read sequencing. *ArXiv12073907 Q-Bio*. Available at: http://arxiv.org/abs/1207.3907.

9. Danecek P, et al. (2011) The variant call format and VCFtools. *Bioinformatics* 27(15):2156–2158.

10. Kawai S, et al. (1992) Drastic alteration of cycloheximide sensitivity by substitution of one amino acid in the L41 ribosomal protein of yeasts. *J Bacteriol* 174(1):254–262.

11. Gruenheit N, et al. (2017) A polychromatic ‘greenbeard’ locus determines patterns of cooperation in a social amoeba. *Nat Commun* 8:14171.

12. Gu Z, Gu L, Eils R, Schlesner M, Brors B (2014) circlize Implements and enhances circular visualization in R. *Bioinforma Oxf Engl* 30(19):2811–2812.

13. Paschke P, et al. (2018) Rapid and efficient genetic engineering of both wild type and axenic strains of *Dictyostelium discoideum*. *PLOS ONE* 13(5):e0196809.

14. Veltman DM, Keizer-Gunnink I, Haastert PJMV (2009) An extrachromosomal, inducible expression system for *Dictyostelium discoideum*. *Plasmid* 61(2):119–125.

15. Davidson AJ, King JS, Insall RH (2013) The use of streptavidin conjugates as immunoblot loading controls and mitochondrial markers for use with *Dictyostelium discoideum*. *BioTechniques* 55(1):39–41.

16. Levi S, Polyakov M, Egelhoff TT (2000) Green fluorescent protein and epitope tag fusion vectors for *Dictyostelium discoideum*. *Plasmid* 44(3):231–238.

17. Fischer M, Haase I, Simmeth E, Gerisch G, Müller-Taubenberger A (2004) A brilliant monomeric red fluorescent protein to visualize cytoskeleton dynamics in *Dictyostelium*. *FEBS Lett* 577(1):227–232.

18. Pang KM, Lynes MA, Knecht DA (1999) Variables controlling the expression level of exogenous genes in *Dictyostelium*. *Plasmid* 41(3):187–197.

19. Bloomfield G, et al. (2015) Neurofibromin controls macropinocytosis and phagocytosis in *Dictyostelium*. *eLife* 4. doi:10.7554/eLife.04940.
